# Evaluation of skeletal muscle microvascular perfusion of lower extremities by cardiovascular magnetic resonance arterial spin labeling, blood oxygenation level-dependent, and intravoxel incoherent motion techniques

**DOI:** 10.1186/s12968-018-0441-3

**Published:** 2018-03-19

**Authors:** Shiteng Suo, Lan Zhang, Hui Tang, Qihong Ni, Suqin Li, Haimin Mao, Xiangyu Liu, Shengyun He, Jianxun Qu, Qing Lu, Jianrong Xu

**Affiliations:** 1 Department of Radiology, Renji Hospital, School of Medicine, Shanghai Jiao Tong University, No. 160, Pujian Rd, Shanghai, 200127 China; 2 Department of Vascular Surgery, Renji Hospital, School of Medicine, Shanghai Jiao Tong University, Shanghai, China; 3 GE Healthcare China, Shanghai, China

**Keywords:** Cardiovascular magnetic resonance, Perfusion, Oxygenation, Blood flow, Diffusion-weighted imaging

## Abstract

**Background:**

Noninvasive cardiovascular magnetic resonance (CMR) techniques including arterial spin labeling (ASL), blood oxygenation level-dependent (BOLD), and intravoxel incoherent motion (IVIM), are capable of measuring tissue perfusion-related parameters. We sought to evaluate and compare these three CMR techniques in characterizing skeletal muscle perfusion in lower extremities and to investigate their abilities to diagnose and assess the severity of peripheral arterial disease (PAD).

**Methods:**

Fifteen healthy young subjects, 14 patients with PAD, and 10 age-matched healthy old subjects underwent ASL, BOLD, and IVIM CMR perfusion imaging. Healthy young and healthy old participants were subjected to a cuff-induced ischemia experiment with pressures of 20 mmHg and 40 mmHg above systolic pressure during imaging. Perfusion-related metrics, including blood flow, T2* relaxation time, perfusion fraction *f,* diffusion coefficient *D*, and pseudodiffusion coefficient D*, were measured in the anterior, lateral, soleus, and gastrocnemius muscle groups. Friedman, Mann-Whitney, Wilcoxon signed rank, and Spearman rank correlation tests were used for statistical analysis.

**Results:**

In cases of significant differences determined by the Friedman test (*P* < 0.05), blood flow, T2*, and *D* values gradually decreased, while *f* values showed a tendency to increase in healthy subjects under cuff compression. No significant correlations were found among the ASL, BOLD, and IVIM parameters (all *P* > 0.05). Blood flow and T2* values showed significant positive correlations with transcutaneous oxygen pressure measurements (ρ = 0.465 and 0.522, respectively; both *P* ≤ 0.001), while *f* values showed a significant negative correlation in healthy young subjects (ρ = − 0.351; *P* = 0.018). T2* was independent of age in every muscle group. T2* values were significantly decreased in PAD patients compared with healthy old subjects and severe PAD patients compared with mild-to-moderate PAD patients (all *P* < 0.0125). Significant correlations were found between T2* and ankle–brachial index values in all muscle groups in PAD patients (ρ = 0.644–0.837; all *P* < 0.0125). Other imaging parameters failed to show benefits towards the diagnosis and disease severity evaluation of PAD.

**Conclusions:**

ASL, BOLD, and IVIM provide complementary information regarding tissue perfusion. Compared with ASL and IVIM, BOLD may be a more reliable technique for assessing PAD in the resting state and could thus be applied together with angiography in clinical studies as a tool to comprehensively assess microvascular and macrovascular properties in PAD patients.

**Electronic supplementary material:**

The online version of this article (10.1186/s12968-018-0441-3) contains supplementary material, which is available to authorized users.

## Background

Peripheral arterial disease (PAD) is a highly prevalent and severe atherosclerotic condition characterized by progressive peripheral arterial development of lower extremeity stenosis/occlusions [1]. Patients affected with PAD suffer from reduced quality of life, and more importantly, increased risk of cardiovascular and cerebrovascular events [2]. Therefore, a noninvasive and objective method is desirable for diagnostic, prognostic, and therapeutic purposes, such as early detection of physiological function changes, clinical risk stratification for predicting myocardial infarction or stroke, and intervention planning for symptomatic patients.

Noninvasive testing of flow-limiting stenosis typically includes measurement of the ankle–brachial index (ABI), the ratio of ankle systolic blood pressure to arm systolic pressure [3]. PAD is considered to be present when the ABI is ≤0.90 and severe when the ABI is ≤0.50 [3]. However, ABI has low sensitivity for PAD diagnosis and may not be necessarily associated with symptom relief after interventions [4, 5]. Transcutaneous oxygen pressure (TcPO2) measurement is an additional method used to indirectly assess the degree of ischemia in ischemic skeletal muscle by measuring tissue oxygenation [6]. The use of TcPO2 measurement is limited because it is confined to the skin and thus does not accurately reflect muscle perfusion [7].

Medical imaging has emerged as an important tool in the diagnosis and management of PAD. Imaging modalities, including computer tomography (CT) angiography, cardiovascular magnetic resonance (CMR) angiography, and digital subtraction angiography, are commonly used to assess abnormal blood vessels and blood flow to the lower extremities. These techniques fail to provide information regarding skeletal muscle microvascular perfusion in the affected extremity [8]. However, as PAD extends beyond the large-vessels, blood flow impairment leads to microvascular dysfunction. Precise assessment of skeletal muscle perfusion would facilitate the comprehensive evaluation of PAD and could be combined with conventional angiography to reveal both functional and anatomical characteristics.

Several CMR techniques can noninvasively measure microvascular perfusion using endogenous tracers, including arterial spin labeling (ASL), blood oxygenation level-dependent (BOLD), and intravoxel incoherent motion (IVIM). ASL magnetically tags arterial blood using radiofrequency pulses, and the perfusion contrast is given by the signal difference between the tagged image and the nontagged control image obtained without net magnetization perturbation in arterial blood [9]. BOLD uses the paramagnetic effect of deoxygenated hemoglobin as an intrinsic contrast agent, which decreases the T2* relaxation signal [10]. IVIM is a variant of conventional diffusion-weighted imaging by separating the effect of blood flow in the randomly oriented capillary network from that of thermally driven water molecular diffusion [11]. ASL, BOLD, and IVIM have been successfully applied to measure skeletal muscle perfusion in previous studies [12–14]. Since perfusion-related metrics derived from these different CMR techniques are based on completely distinct mechanisms, they may depict different aspects of muscle perfusion properties. ASL is more related to the function of blood delivery to target tissues, BOLD is related to tissue oxygenation, and IVIM is related to pseudodiffusion within capillary beds. We hypothesize that multi-parametric CMR techniques, including ASL, BOLD, and IVIM, could provide complementary information regarding perfusion in skeletal muscles and would represent various alteration patterns in the presence of perfusion deficits.

Hence, this study aimed to 1) test the feasibility of using ASL, BOLD, and IVIM to measure perfusion changes in the lower extremities of healthy subjects under different external compression statuses; 2) validate the associations between ASL-, BOLD-, and IVIM-derived parameters and TcPO2 measurements; 3) evaluate the effects of age on imaging parameter measurements of ASL, BOLD, and IVIM in healthy subjects at rest; 4) use ASL, BOLD, and IVIM to compare perfusion in affected and contralateral (asymptomatic) lower extremities in PAD patients at rest; 5) compare the capabilities of resting-state ASL, BOLD, and IVIM in detecting perfusion differences between PAD patients and age-matched healthy subjects and between mild-to-moderate and severe PAD patients; and 6) investigate the associations between ASL-, BOLD-, and IVIM-derived parameters and ABI in PAD patients.

## Methods

The local institutional review board approved the prospective study, and written consent was obtained from all subjects prior to participation. Technical support for imaging sequence optimization was provided by a GE Healthcare employee (JQ). Authors not associated with GE Healthcare had full control of the data and information submitted for publication.

### Subjects

Between February 2016 and October 2017, three groups of subjects were enrolled: 1) healthy young subjects (*n* = 15); 2) PAD patients (*n* = 14); and 3) age-matched healthy old subjects (*n* = 10). None of the healthy subjects showed clinical evidence of PAD, cardiac insufficiency, hypoxic pulmonary diseases, or lower extremity venous disorders. Each of these subjects had a normal peripheral pulse status and an ABI > 0.90, and they were all non-smokers. The healthy old group was age-matched to the PAD group to eliminate the confounding effect of age on CMR perfusion parameter measurements. Patients with PAD were recruited from the department of vascular surgery with symptoms of intermittent claudication, rest pain, or critical limb ischemia and an ABI ≤ 0.90. The PAD group was then stratified into two disease severity subgroups based on ABI: 1) the mild-to-moderate group corresponded to an ABI of 0.51 to 0.90 (*n* = 7); and 2) the severe group corresponded to an ABI ≤ 0.50 (n = 7). Details for each group are listed in Table 1.

**Table 1:** Demographics

Subjects	Number	Sex (M/F)	Age	ABI	Hypertension	Diabetes mellitus
Healthy Young	15	7/8	24 (20–28)	All ABI > 1	0	0
Healthy Old	10	6/4	60 (50–67)	1.09 (0.96–1.22)	1	0
PAD	14	9/5	65 (55–79)	0.52 (0.00–0.90)	10	8
Mild-to-moderate	7	4/3	59 (55–66)	0.65 (0.55–0.90)	5	5
Severe	7	5/2	69 (57–79)	0.35 (0.00–0.48)	5	3

Data are medians with ranges in parentheses or n

*ABI* ankle-brachial index, *PAD* peripheral arterial disease

### Imaging protocol

**Fig. 1:**
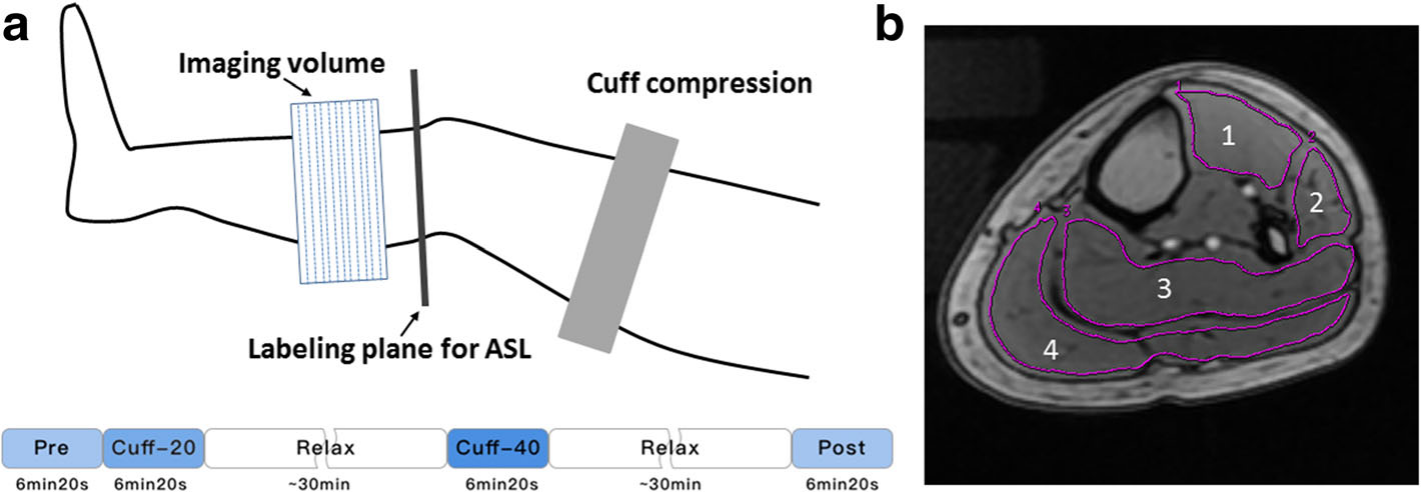
CMR imaging workflow. **a** Ischemia via arterial occlusion was induced in the lower extremity with an occlusive cuff tied around the middle of one thigh. The axial image slice was acquired at the widest part of the lower extremity. Four experimental statuses are defined as follows: baseline (Pre), cuff compression with a pressure of 20 mmHg above systolic pressure (Cuff-20), cuff compression with a pressure of 40 mmHg above systolic pressure (Cuff-40) and recovery period (Post). **b** T1-weighted anatomical images allow for the accurate delineation of muscle groups (1 = anterior, 2 = lateral, 3 = soleus, and 4 = gastrocnemius) in the lower extremity

In preparation for the scans, all subjects were asked to refrain from alcohol, caffeine, and vigorous exercise 12 h before imaging. The healthy young and healthy old subjects were subjected to a cuff-induced arterial occlusion experiment during the CMR imaging scans to test the sensitivities of ASL, BOLD, and IVIM to pressure variations. Ischemia via arterial occlusion was induced in the lower extremity by a sphygmomanometer cuff tied around the middle of one thigh. The contralateral lower extremity without intervention was imaged simultaneously as the control side. ASL, BOLD, and IVIM were conducted four times in the following order: baseline (Pre), cuff compression with a pressure of 20 mmHg above systolic pressure (Cuff-20), cuff compression with a pressure of 40 mmHg above systolic pressure (Cuff-40), and recovery period (Post). Two different cuff pressures were used to verify whether different air pressures could modulate the perfusion signal intensity and thus provoke different degrees of ischemia. The pressure of 40 mmHg above systolic pressure was chosen in accordance with the previously reported range of 30–50 mmHg above systolic pressure, which is recommended for provoking complete and reproducible ischemia [15]. During the Cuff-20 and Cuff-40 sessions, the cuff was kept inflated until all scans were completed (6 min 20 s). The last three sessions were performed at 30-min intervals to avoid the interference from the preceding session (Fig. 1). To test the interscan reproducibility, 5 healthy subjects were subjected to a second scan within 1 week. Patients with PAD received baseline examinations only at rest. All subjects were asked to lie still in the supine position for approximately 15 min before the onset of CMR imaging to ensure that their legs were at heart height [16], and remain still during the entire examination.

All the CMR measurements were carried out on a 3-T CMR system (HDxt, General Electric Healthcare, Waukesha, Wisconsin, USA) with an eight-channel cardiac coil. The subjects were investigated in the supine position. Prior to acquisition of the CMR perfusion images, axial three-dimensional (3D) spoiled gradient-recalled echo T1-weighted images (repetition time ms/echo time ms 4.1/1.5; flip angle = 12°; matrix, 320 × 320; FOV, 32 × 32 mm^2^; slice thickness = 5 mm; number of slices = 12) were acquired for use as anatomical landmarks. A FOV of 32 × 32 mm^2^ could cover both legs without leaving anything outside, thus prohibiting wrapping artifacts [17]. Pseudocontinuous ASL was performed using an interleaved 3D stack of spiral fast spin-echo sequence with background suppression. Each spiral arm included 512 sampling points in k-space and a total of 6 spiral arms were acquired. Background suppression was achieved via 5 inversion pulses placed 0, 1465, 2100, 2600, and 2880 ms after the labeling start point to suppress a broad range of T1 values [18]. Other ASL parameters were as follows: repetition time ms/echo time ms = 4316/9.4; bandwidth = 62.5 kHz; FOV = 32 × 32 mm^2^; slice thickness = 5 mm; number of slices = 12; number of averages = 2; post-labeling delay time = 1525 ms. A post-labeling delay time of 1525 ms before imaging was used to allow the blood to perfuse all muscle groups, which was similar to that used in a previous study [19]. BOLD was performed using a multi-echo gradient-recalled echo sequence implementing the following parameters: repetition time ms/echo times ms 875/(2.5, 6.7, 10.9, 15.1, 19.3, 23.5, 27.7, 31.9, 36.1, 40.3, 44.5, 48.7, 52.9, 57.1, 61.3, and 65.5); matrix = 256 × 128; FOV = 32 × 32 mm^2^; slice thickness/gap = 5/0 mm; number of slices = 12. IVIM imaging was performed using a single shot spin-echo echo-planar imaging sequence at 9 *b*-values (0, 20, 50, 100, 150, 200, 300, 500, and 800 s/mm^2^) in three orthogonal gradient directions, with the following parameters: repetition time ms/echo time ms = 2800/70; matrix = 192 × 192; FOV = 32 × 32 mm^2^; slice thickness/gap = 5/0 mm; number of slices = 12; number of averages = 3; parallel imaging factor = two. A standard monopolar Stejskal-Tanner diffusion encoding scheme was applied with diffusion gradient pulse duration of 16 ms. The acquisition times for ASL, BOLD, and IVIM were 2 min 18 s, 1 min 59 s, and 2 min 3 s, respectively. All imaging was performed in the axial plane at the level of the middle calf.

### Image analysis

ASL, BOLD, and IVIM data were post-processed on a pixel-by-pixel basis on a workstation (Advantage Workstation 4.5; General Electric Healthcare) to obtain corresponding parametric maps of blood flow, T2* relaxation time, perfusion fraction *f*, diffusion coefficient *D*, and pseudodiffusion coefficient D*.

Pseudocontinuous ASL perfusion was calculated using a one-compartment model for blood after subtracting the tagged images from the nontagged control images. Blood flow was quantified using the following equation:

$$ BF=\frac{\lambda \cdot \left({SI}_{control}-{SI}_{label}\right)\cdot {e}^{\frac{PLD}{T_{1, blood}}}}{2\cdot \alpha \cdot {T}_{1, blood}\cdot {SI}_{PD}\cdot \left(1-{e}^{-\frac{\tau }{T_{1, blood}}}\right)}, $$

where *PLD* is the post-labeling delay (1525 ms), *λ* is the tissue partition coefficient (0.9 ml/g) [19], *T*_1, *blood*_ is the longitudinal relaxation time of blood (1600 ms) [19], *α* is the labeling efficiency (0.80 × 0.75) (label pseudocontinuous ASL × background suppression) [18], *τ* is the labeling duration (1450 ms), *SI*_*PD*_ is the proton density reference without labeling or background saturation, and *SI*_*control*_ and *SI*_*label*_ are the control and tagged signals, respectively. The arterial transit time was ignored in the quantification.

T2* relaxation time was calculated from the multi-echo T2* gradient-recalled echo data using the least-square fit of monoexponential decay [20], according to the following equation: S(TE) = S_TE0_·exp.(−TE/T2*), where TE is the gradient echo time and S(TE) and S_TE0_ are the measured signal intensities for TE ≠ 0 and TE = 0, respectively.

The multi-*b*-value diffusion-weighted images were analyzed using the IVIM model according to the following equation: S(*b*) = S_*b*0_·[*f*·exp.(−*b*D*) + (1-*f*) ·exp.(−*b*D)], where S(*b*) is the measured signal intensity obtained with a nonzero *b*-value and S_*b*0_ is the measured signal intensity for *b* = 0. With this equation, perfusion fraction *f* together with diffusion coefficient *D* and pseudodiffusion coefficient D* were calculated using a nonlinear biexponential fit based on the Levenberg-Marquardt technique [21].

For BOLD and IVIM analysis, goodness of fit was assessed using *R*^2^ = 1 − *ESS*/*TSS*, where *ESS* is the sum of squared errors between the data points and the fitting curve and *TSS* is the sum of squared differences between the data points and the mean value of all data points. Additional computations were performed to assess the signal-to-noise ratios (SNRs) of the images obtained with TE of 65.5 ms for BOLD and *b*-value of 800 s/mm^2^ for IVIM. Noisy images were excluded from curve fitting.

Regions of interest (ROIs) were manually drawn on T1-weighted images around the 4 muscle groups (anterior, lateral, soleus, and gastrocnemius) at the largest cross-sectional area of the calf on both the experimental and control sides (Fig. 1). Attention was given to exclude areas influenced by bones and large vessels, and the inter-osseous muscle was not investigated because it contains a relatively large number of vessels [22]. ROIs were then copied and pasted into the corresponding functional perfusion maps. Average values within ROIs were recorded. Independent analysis of perfusion maps (from 7 randomly selected healthy subjects) by 2 radiologists blinded to the clinical outcomes was conducted to evaluate the interreader reproducibility. In addition, the repeat scans of 5 healthy subjects were analyzed by the same blinded radiologist to test the interscan reproducibility. Normalized values were obtained by dividing each imaging parameter value obtained under the experimental statuses (Cuff-20/Cuff-40/Post) by the baseline measurement (Pre).

### TcPO2 measurements

TcPO2 measurements were acquired in all healthy young subjects the day after the CMR examinations using a TcPO2 monitoring system (Periflux System 5000; Perimed, Jarfalla, Sweden) in an air-conditioned room maintained at 22 °C. The cuff-induced ischemia paradigm followed the same process as that described for the CMR experiments. One author (LZ, 12 years of experience in TcPO2 measurement) placed the electrode at the same spot at which the CMR measurements were taken (i.e., the medial upper third of the calf adjacent to the gastrocnemius muscle at the maximal calf diameter) [7].

### Statistical analysis

Statistical analyses were performed using SPSS version 20 (International Business Machines, Armonk, New York, USA), OriginPro 2016 (OriginLab Corp., Northampton, Massachusetts, USA), and Prism 5 (GraphPad Software Inc., La Jolla, California, USA). *P*-values less than 0.05 were considered to indicate statistical significance.

All data were expressed as the median (range) owing to non-normal data distributions. To assess interreader and interscan reproducibility, the intraclass correlation coefficient (ICC) was calculated. ICC values less than 0.40 indicated poor reproducibility, those ranging from 0.40 to 0.75 indicated fair to good reproducibility, and those higher than 0.75 indicated excellent reproducibility.

To determine whether differences existed in each imaging parameter value under different statuses (Pre/Cuff-20/Cuff-40/Post) in the experimental and control lower extremities, the Friedman test was used. In cases of statistical significance, further pairwise comparisons with the Dunn test were performed. The Wilcoxon signed rank test was used to compare imaging parameter measurements between left and right lower extremities in all healthy subjects under different statuses. The non-parametric Spearman rank correlation test was performed to assess correlations between imaging parameters derived from different methods, as well as between imaging parameters in gastrocnemius and TcPO2 measurements. Strength of correlation based on the Spearman rank correlation coefficient (ρ) was interpreted as follows: 0.00 to 0.20, very weak to negligible correlation; 0.21 to 0.40, weak correlation; 0.41 to 0.70, moderate correlation; 0.71 to 0.90, strong correlation; and 0.91 to 1.00, very strong correlation [23]. The effects of age on the baseline imaging parameter measurements were investigated by comparing the values between the healthy young and healthy old groups with the Mann-Whitney *U* test. The Wilcoxon signed rank test was used for comparisons of imaging parameter measurements between the left and right lower extremities in PAD patients. Then, imaging parameters were compared between PAD patients and healthy old subjects, as well as between mild-to-moderate and severe PAD patients using the Mann-Whitney *U* test. Finally, correlations between imaging parameters and ABI in PAD patients were assessed using the Spearman rank correlation test. Bonferroni correction for multiple comparisons for the number of muscle groups was applied when necessary.

The sample size of patients included in this study was estimated using one-sided calculations with α of 0.05 and a power of 80% to detect an absolute T2* decrease of 4 ms (compared with normal) with a standard deviation of 2 ms based on the results of previous studies [24, 25]. Assuming a 20% dropout rate, it was determined that 10 participants were required.

## Results

All subjects successfully completed the CMR examinations (Table 1). Cuff compression of the thigh was well tolerated. Quantitative image analysis was conducted for each participant. Representative source images and parametric maps are illustrated in Additional file 1: Figure S1. The median *R*^*2*^ values for BOLD and IVIM fittings were 0.90 (range, 0.80–0.98) and 0.98 (range, 0.92–1.00), respectively. The median SNR values on the TE = 65.5 ms and *b* = 800 s/mm^2^ images were 19.2 (range, 14.4–23.5) and 17.2 (range, 14.2–32.0), respectively. The overall interreader reproducibility was fair to excellent, with ICC values of 0.83 for blood flow, 0.93 for T2*, 0.77 for *f,* 0.92 for *D*, and 0.64 for D*. The overall interscan reproducibility was fair to excellent, with ICC values of 0.73 for blood flow, 0.85 for T2*, 0.67 for *f,* 0.84 for *D*, and 0.55 for D*.

### Functional imaging parameter variation under the cuff compression paradigm

**Fig. 2:**
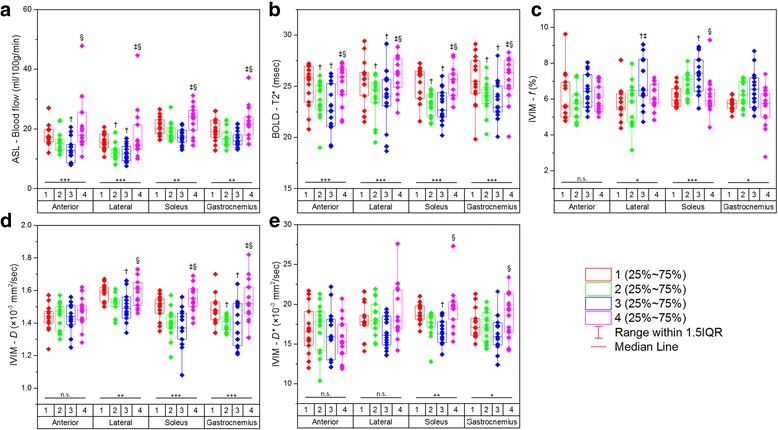
Graphs presenting serial measurements of imaging parameters from ASL (**a**), BOLD (**b**), and IVIM (**c, d, e**) for the anterior, lateral, soleus, and gastrocnemius muscle groups in healthy young subjects. Comparisons among all four statuses (1 = baseline; 2 = 20-mmHg cuff compression; 3 = 40-mmHg cuff compression; 4 = recovery) for every muscle group were performed using the Friedman test (n.s. = not significant; * = *P* < 0.05; ** = *P* < 0.01; *** = *P* < 0.001). Further pairwise comparisons were made with the Dunn test († = *P* < 0.05 compared with baseline; ‡ = *P* < 0.05 compared with 20-mmHg cuff compression; § = *P* < 0.05 compared with 40-mmHg cuff compression)

**Fig. 3:**
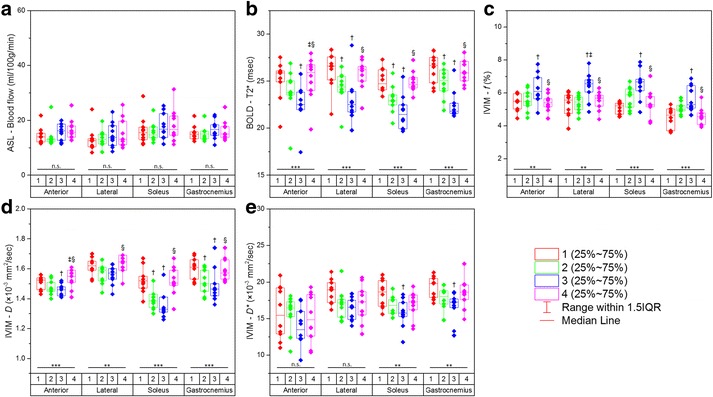
Graphs presenting serial measurements of imaging parameters from ASL (**a**), BOLD (**b**), and IVIM (**c, d, e**) for the anterior, lateral, soleus, and gastrocnemius muscle groups in healthy old subjects. Comparisons among all four statuses (1 = baseline; 2 = 20-mmHg cuff compression; 3 = 40-mmHg cuff compression; 4 = recovery) for every muscle group were performed using the Friedman test (n.s. = not significant; * = *P* < 0.05; ** = *P* < 0.01; *** = *P* < 0.001). Further pairwise comparisons were made with the Dunn test († = *P* < 0.05 compared with baseline; ‡ = *P* < 0.05 compared with 20-mmHg cuff compression; § = *P* < 0.05 compared with 40-mmHg cuff compression)

Changes in quantitative imaging parameters under the cuff compression paradigm in the healthy young and healthy old groups are illustrated in Figs. 2 and 3 for the experimental lower extremity and in Additional file 2: Figure S2 and Additional file 3: Figure S3 for the control side.

In the healthy young group on the experimental side, only blood flow and T2* values showed significant differences among the 4 statuses in all muscle groups (all *P* < 0.01 for blood flow, and all *P* < 0.001 for T2*). *f* and *D* values showed significant differences in the lateral, soleus, and gastrocnemius muscle groups, and D* values were significantly different in the soleus and gastrocnemius muscle groups (all *P* < 0.05). Under the Cuff-20/Cuff-40 compression statuses, T2* values were significantly lower than the baseline measurements in all muscle groups (all *P* < 0.05), and blood flow, *f*, *D*, and D* values showed marked differences in only some muscle groups. No significant differences for any parameter in either muscle group were observed between the Post and Pre statuses (all *P* > 0.05) (Fig. 2).

In the healthy old group on the experimental side, only T2*, *f*, and *D* values showed significant differences among the 4 statuses in all muscle groups (all *P* < 0.001 for T2*, and all *P* < 0.01 for *f* and *D*). D* values showed significant differences in the soleus and gastrocnemius muscle groups (both *P* < 0.01). Blood flow values did not significantly differ in any muscle group (all *P* > 0.05). Under the Cuff-20/Cuff-40 compression statuses, significant differences were found for T2* values compared with those at baseline in all muscle groups except for the anterior muscle group under the 20-mmHg status (all *P* < 0.05)*.* Furthermore, *f*, *D*, and D* values were significantly different in several muscle groups under these statuses (all *P* < 0.05). Additionally, no significant differences between Pre and Post measurements were observed for any of the parameters (all *P* > 0.05) (Fig. 3).

In both the healthy young and healthy old groups on the experimental side, in the cases of significant differences, blood flow, T2*, and *D* values gradually decreased under the Cuff-20 and Cuff-40 compression statuses, while *f* values showed a tendency to increase. During the recovery period, all parameters nearly returned to normal (Figs. 2 and 3).

In both the healthy young and healthy old groups on the control side, among all parameters, only T2* values in some muscle groups showed significant differences among the 4 statuses (*P* < 0.05) (Additional file 2: Figure S2 and Additional file 3: Figure S3).

### Comparison of imaging parameters between the experimental and control sides

Results of Wilcoxon signed rank test comparing functional imaging parameters between the experimental and control sides under different statuses are illustrated in Table 2. No significant differences in any parameter were observed between the left and right lower extremities in healthy subjects at rest (all *P* > 0.0125). Most muscle groups exhibited significant differences in perfusion-related parameter values especially when the cuff compression pressure was increased to 40 mmHg above systolic pressure.

**Table 2:** Comparison of functional perfusion parameters between experimental and control sides under different statuses in healthy subjects

Parameter	Test	*P*-value			
		Anterior	Lateral	Soleus	Gastrocnemius
ASL-Blood flow	Pre	0.040	0.779	0.657	0.819
	Cuff-20	0.619	0.104	0.074	**0.011**
	Cuff-40	0.545	0.026	**0.006**	**0.004**
	Post	0.020	0.339	0.026	0.088
BOLD-T2*	Pre	0.093	0.135	0.040	0.710
	Cuff-20	0.946	0.030	**< 0.001**	**0.007**
	Cuff-40	**0.009**	**0.001**	**< 0.001**	**< 0.001**
	Post	0.023	0.051	**0.007**	0.015
IVIM-*f*	Pre	0.036	0.104	0.853	0.767
	Cuff-20	0.231	0.333	0.018	0.059
	Cuff-40	**0.003**	0.023	**< 0.001**	**0.001**
	Post	0.363	0.786	0.757	0.216
IVIM-*D*	Pre	0.909	0.057	0.840	0.449
	Cuff-20	0.543	0.440	**0.002**	**0.005**
	Cuff-40	0.483	0.495	**< 0.001**	**0.002**
	Post	0.078	**0.001**	**0.007**	**0.004**
IVIM-D*	Pre	0.648	0.407	0.872	0.553
	Cuff-20	0.590	0.313	**0.011**	0.027
	Cuff-40	0.427	**0.003**	**0.001**	**0.002**
	Post	0.174	0.338	0.607	0.288

Data are *P-*values derived from the Wilcoxon signed rank test. After Bonferroni correction, *P* < 0.0125 denotes statistical significance. The statistically significant values are presented in bold

### Correlation between functional imaging parameters derived from ASL, BOLD, and IVIM

No significant correlations between functional imaging parameters derived from the different methods were observed (all *P* > 0.05) (Additional file 4: Table S1).

### Correlation between functional imaging parameters and TcPO2 measurements

**Fig. 4:**
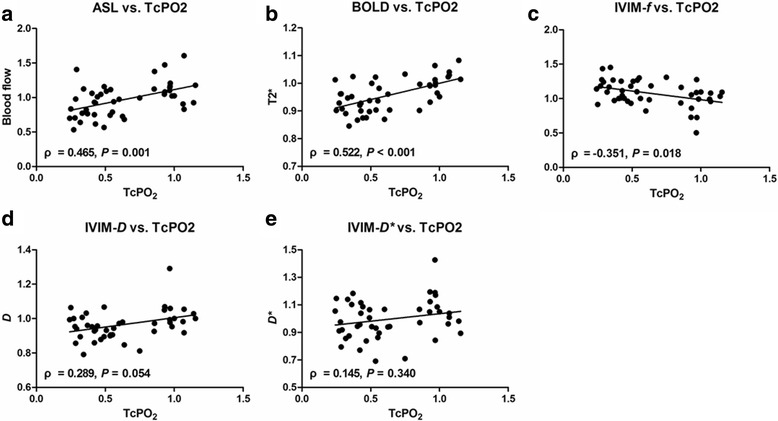
Graphs depicting relationships between normalized CMR imaging parameters of blood flow (**a**), T2* (**b**), *f* (**c**), *D* (**d**), and D* (**e**) and normalized TcPO2 measurements. Significant correlations were observed for (**a**), (**b**), and (**c**), with Spearman rank correlation coefficients (ρ) of 0.465, 0.522, and − 0.351, respectively

The normalized CMR imaging parameters blood flow, T2*, and *f* were all correlated with normalized TcPO2 measurements (Fig. 4). Blood flow and T2* showed significant moderate correlations with TcPO2 measurements (ρ = 0.465 (*P* = 0.001) and ρ = 0.522 (*P* < 0.001), respectively). A significant negative correlation was observed between *f* and TcPO2 measurements (*P* = 0.018), although the correlation was weak (ρ = − 0.351). No significant correlation was found for *D* (*P* = 0.054) or D* values (*P* = 0.340).

### Effects of age on resting-state functional imaging parameter measurements in healthy subjects

Within the muscle groups studied, blood flow for all muscle groups, *f* for the soleus and gastrocnemius groups, and *D* and D* for the gastrocnemius group demonstrated significant differences between the healthy young and healthy old groups at rest (all *P* < 0.0125). Only T2* was found to be independent of age in every muscle group (all *P* > 0.0125) (Table 3).

**Table 3:** Comparison of functional imaging parameters between healthy young and healthy old subjects

Parameter	*P-*value			
	Anterior	Lateral	Soleus	Gastrocnemius
ASL-Blood flow	**0.005**	**0.007**	**0.004**	**0.003**
BOLD-T2*	0.978	0.367	0.531	0.567
IVIM-*f*	0.216	0.285	**< 0.001**	**< 0.001**
IVIM-*D*	0.014	0.160	0.807	**0.012**
IVIM-D*	0.567	0.338	0.683	**0.011**

Data are *P-*values derived from the Mann-Whitney *U* test. After Bonferroni correction, *P* < 0.0125 denotes statistical significance. The statistically significant values are presented in bold

*ASL* arterial spin labeling, *BOLD* blood oxygenation level-dependent, *IVIM* intravoxel incoherent motion

### Comparison of resting-state functional imaging parameters between the affected and contralateral sides in PAD patients

In PAD patients, T2* was markedly reduced on the affected side compared with that on the contralateral side; significance was reached in the anterior muscle group (*P* = 0.005), and no significance was reached in the lateral, soleus, or gastrocnemius muscle groups (all *P* > 0.0125) (Additional file 5: Table S2).

### Using ASL, BOLD, and IVIM to measure perfusion in PAD patients

Results of Mann-Whitney *U* tests comparing functional perfusion parameters between PAD patients and age-matched healthy subjects and between mild-to-moderate and severe PAD patients at rest are illustrated in Table 4. Among all parameters, T2* showed the best performance for the discrimination, with significantly reduced values observed in PAD patients compared with age-matched healthy old subjects and severe PAD patients compared with mild-to-moderate patients in all muscle groups (all *P* < 0.0125). No significant differences in other parameters in any muscle group were observed except D* values between PAD patients and healthy old subjects in the lateral muscle group.

**Table 4:** Associations of the presence and severity of PAD with functional imaging parameters

Parameter		AHO vs. PAD			Mild-to-moderate vs. Severe PAD		
		AHO	PAD	*P*-value	Mild-to-moderate	Severe	*P*-value
ASL-Blood flow (ml/100 g/min)	Anterior	14.0 (11.6–21.8)	15.2 (8.1–34.2)	0.666	16.3 (9.9–23.7)	14.5 (8.1–34.2)	0.318
	Lateral	12.6 (8.3–24.0)	12.9 (8.2–34.0)	0.886	13.7 (8.2–19.7)	12.0 (9.2–34.0)	0.805
	Soleus	15.5 (11.3–28.8)	14.7 (12.0–40.1)	0.796	16.5 (12.0–26.7)	14.2 (12.3–40.1)	0.456
	Gastrocnemius	14.9 (12.4–21.6)	14.5 (10.5–25.5)	0.625	14.4 (11.0–19.0)	14.8 (10.5–25.5)	0.805
BOLD-T2* (ms)	Anterior	25.9 (20.2–27.5)	21.9 (13.4–26.8)	**0.002**	22.3 (21.7–26.8)	19.4 (13.4–23.5)	**0.011**
	Lateral	26.6 (21.5–28.3)	21.8 (11.5–28.7)	**0.011**	25.2 (19.9–28.7)	16.6 (11.5–22.9)	**0.004**
	Soleus	24.7 (23.4–27.3)	22.5 (14.8–26.6)	**0.007**	24.3 (22.1–26.6)	17.4 (14.8–22.8)	**0.001**
	Gastrocnemius	26.7 (23.9–28.3)	22.5 (13.5–26.1)	**0.001**	24.7 (20.2–26.1)	19.3 (13.5–24.8)	**0.011**
IVIM-*f* (%)	Anterior	5.5 (4.5–6.0)	5.7 (4.5–12.0)	0.235	5.7 (4.5–12.0)	6.6 (5.2–7.7)	0.383
	Lateral	5.6 (3.8–6.1)	5.6 (4.4–14.5)	0.371	4.8 (4.4–14.5)	5.7 (5.1–8.8)	0.535
	Soleus	5.2 (4.6–5.5)	5.2 (3.7–14.2)	0.472	4.9 (3.7–14.2)	5.4 (5.0–6.7)	0.456
	Gastrocnemius	4.7 (3.6–5.3)	5.6 (3.7–7.7)	0.016	5.8 (3.7–7.7)	5.4 (4.3–5.8)	0.209
IVIM-*D* (×10^−3^ mm^2^ /s)	Anterior	1.53 (1.43–1.56)	1.48 (1.24–2.02)	0.122	1.54 (1.24–2.02)	1.38 (1.33–1.52)	0.053
	Lateral	1.62 (1.52–1.70)	1.51 (0.97–1.72)	0.186	1.65 (0.97–1.72)	1.48 (1.18–1.55)	0.165
	Soleus	1.52 (1.38–1.67)	1.44 (1.28–1.99)	0.235	1.47 (1.42–1.99)	1.32 (1.28–1.58)	0.038
	Gastrocnemius	1.61 (1.50–1.70)	1.51 (1.33–1.88)	0.371	1.61 (1.45–1.88)	1.40 (1.33–1.74)	0.073
IVIM-D* (×10^−3^ mm^2^ /s)	Anterior	15.5 (11.0–20.9)	17.2 (11.0–20.5)	0.437	17.4 (11.0–20.5)	17.0 (14.9–19.9)	0.620
	Lateral	19.0 (16.2–21.4)	14.8 (9.1–18.1)	**0.001**	14.2 (9.1–17.7)	17.3 (10.6–18.1)	0.318
	Soleus	18.7 (15.6–21.0)	17.2 (9.4–27.5)	0.235	17.1 (9.4–21.8)	17.3 (14.4–27.5)	0.902
	Gastrocnemius	19.8 (17.1–21.3)	17.3 (12.5–21.9)	0.391	17.6 (12.5–21.8)	16.4 (14.3–21.9)	0.902

Data are medians with ranges in parentheses. After Bonferroni correction, *P* < 0.0125 denotes statistical significance. The statistically significant values are presented in bold

*AHO* age-matched healthy old subjects, *PAD* peripheral arterial disease, *ASL*arterial spin labeling, *BOLD* blood oxygenation level-dependent, *IVIM* intravoxel incoherent motion

**Fig. 5:**
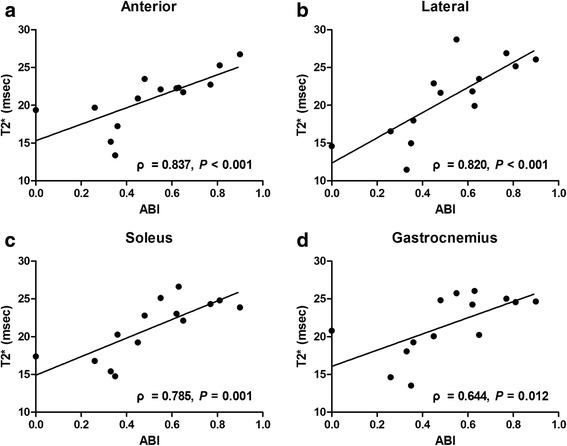
Graphs depicting relationships between T2* in the anterior (**a**), lateral (**b**), soleus (**c**), and gastrocnemius (**d**) muscle groups and ABI in PAD patients. Significant correlations were observed for all muscle groups, with Spearman rank correlation coefficients (ρ) of 0.837, 0.820, 0.785, and 0.644, respectively

Spearman rank correlation analysis showed that in PAD patients, T2* was significantly correlated with ABI in the anterior (ρ = 0.837; *P* < 0.001), lateral (ρ = 0.820; *P* < 0.001), soleus (ρ = 0.785; *P* = 0.001), and gastrocnemius (ρ = 0.644; *P* = 0.012) muscle groups (Fig. 5), whereas no significant correlation was observed for the other parameters (all *P* > 0.05).

## Discussion

Noninvasive monitoring of skeletal muscle perfusion in the lower extremities is critical for PAD patient management as perfusion can provide insight into microvascular function and endothelial integrity [5]. Advanced CMR techniques, including ASL, BOLD, and IVIM, have been utilized in the assessment of skeletal muscle perfusion [12–14]; however, to our knowledge, few studies have been performed to directly compare these techniques in the same subject cohort [5, 26]. Furthermore, there is limited literature on comparisons of these CMR parameters with routinely used parameters in clinical practice such as TcPO2 and ABI [5, 7, 13]. The results of our study suggested that 1) ASL, BOLD, and IVIM could respond to cuff-induced ischemia in healthy subjects—that is, when the difference reached a significant level, ASL-derived blood flow values, BOLD-derived T2* values, and IVIM-derived *D* values tended to decrease with increasing external pressure while IVIM-derived *f* values tended to increase under cuff compression; 2) blood flow, T2*, and *f* values were all correlated with TcPO2 measurements; 3) ASL of all muscle groups and IVIM of the gastrocnemius group were influenced by age; only BOLD was independent of age in every muscle group; 4) BOLD could detect perfusion differences between the affected and contralateral lower extremities in PAD patients; 5) BOLD could separate PAD patients from healthy old subjects and PAD patients with different severities; and 6) BOLD-derived T2* was correlated with ABI in PAD patients.

Similar to the results in published literature [19, 27], baseline blood flow in skeletal muscle as measured by ASL in the lower extremity was mostly near or less than 20 ml/100 g/min in our study. For BOLD CMR, we observed a baseline mean T2* value of approximately 25 ms in healthy subjects, which lies within the previously reported range of 22–27 ms [13, 25, 28, 29]. IVIM imaging of skeletal muscle in the lower extremity has rarely been studied. In other parts of the body, IVIM-derived *f*, *D*, and D* values were reported to be 3%, 1.45 × 10^− 3^ mm^2^/s, and 28.5 × 10^− 3^ mm^2^/s, respectively, in the forearm muscle at rest by Filli et al. [14], and 6.6%, 1.45 × 10^− 3^ mm^2^/s, and 11.7× 10^− 3^ mm^2^/s, respectively, in the shoulder muscle by Nguyen et al. [30]. These findings are consistent with our observations (e.g., 5.9%, 1.52 × 10^− 3^ mm^2^/s, and 18.6 × 10^− 3^ mm^2^/s, respectively, in the soleus muscle in healthy young subjects).

Under cuff compression conditions, negative ASL and BOLD contrasts in healthy young subjects developed due to ischemic insult, which agreed with previous studies [7, 12, 13, 25, 31]. ASL is capable of measuring blood flow through muscle tissue microvasculature given that ASL and radiolabeled microsphere measurements in rat leg muscle have shown good correlation for perfusion [32]. Cuff compression interrupted both arterial inflow and venous outflow simultaneously, thus provoking reduced blood flow obtained by ASL. Although the exact source of the BOLD signal in skeletal muscle is not yet fully understood, it is generally accepted that the signal is primarily associated with capillary and blood oxygenation state [7]. Lebon et al. also found that the T2* signal in muscle rapidly decreased during ischemia and attributed this change to early hemoglobin desaturation [33]. This finding is logical given that the BOLD signal changed almost synchronously with hemoglobin desaturation but preceded myoglobin desaturation [33], as the dissociation constant of hemoglobin is more than 10 times higher than that of myoglobin. IVIM-derived *D* values also showed a decreasing trend under cuff-induced ischemic conditions. Local ischemia leads to decreased diffusivity of water molecules within muscles, and this decreased *D* was mostly likely attributed to this physical effect. Conversely, the perfusion fraction *f* derived from IVIM showed a tendency to increase in the case of arterial occlusion. Given that *f* reflects the signal fraction of capillary blood flow in entire water molecule diffusion pool within each voxel [11], it can be hypothesized that obstruction of venous reflux is probably responsible for the altered *f* values. In addition, it has been suggested that decrease in venous oxygen saturation may release relaxing factors than can cause microvascular dilation [34], which may also increase the *f* value. In healthy old subjects, changing trends for the imaging parameters were similar to those observed in the healthy young group except for ASL. ASL is limited by the intrinsic low SNR in skeletal muscle, wherein the ASL signal represents only 0.5%–1% of the raw image intensity [8]. This notion may account for the lack of statistical significance of ASL measurements, especially given that the healthy old sample number was small (*n* = 10).

Interestingly, we also observed aberrant T2* signal changes on the control sides of healthy subjects during the cuff compression experiment on the other lower extremity, in accordance with the findings of a previous study [35]. Yeung et al. suggested that this might be because of the high sensitivity of BOLD CMR imaging to local magnetic field disturbances caused by magnetic susceptibility effects, which may be induced by oxygen in the air at high pressure during cuff inflation [35].

Ledermann et al. reported that BOLD CMR imaging correlated with TcPO2 measurements in healthy volunteers during muscle ischemia [7], which was consistent with our results. However, the correlation observed by Ledermann et al. was stronger (correlation coefficient, 0.96) than that in our study (correlation coefficient, 0.522), primarily because Ledermann et al. averaged signal intensities across all volunteers for statistics, not for individuals. In addition, ASL-derived blood flow and IVIM-derived *f* values were also found to correlate with TcPO2 measurements in our study, with correlation coefficients of 0.465 and − 0.351, respectively. The correlation between BOLD-derived T2* and TcPO2 measurements was stronger than those between the other parameters, which may be attributed to the fact that both BOLD CMR imaging and TcPO2 examination are directly associated with the oxygenation state at the microvascular level.

Age effects on parameter measurements varied among the different sequences and muscles. In our study, no significant differences between the healthy young and healthy old groups were observed for the baseline T2* value, indicating that age did not appear to affect BOLD in healthy subjects at rest. This finding was in accordance with other observations [28]. In contrast, ASL and IVIM were more easily influenced by the age factor especially in the gastrocnemius muscle group. The gastrocnemius is a fast-twitch type muscle, whereas the soleus belongs to the slow-twitch type. Degenerative processes of muscle fibers have been demonstrated to differ with fiber type, and the fast-twitch muscle is more prone to aging and fatigue [36, 37].

The ABI is a measure providing objective data for diagnosing PAD. When applying these imaging techniques on PAD patients, we found that BOLD was capable of detecting perfusion deficits at rest better than ASL and IVIM. Lower T2* values were related to the presence of PAD and more disease severity stratified by ABI. As discussed earlier, BOLD effect is generally assumed to reflect blood oxygenation state influenced by the ratio of oxygenated to deoxygenated hemoglobin, which is determined by the balance between oxygen supply and consumption [15]. In PAD, arterial blood flow in the lower extremities is limited, leading to reduced oxygenated hemoglobin. Moreover, the impaired vascular function causes a longer contact time between blood and myocytes, leading to more efficient deoxygenation of hemoglobin [15]. These two effects both contribute to a reduced T2* value. In a previous study by Englund et al., BOLD-derived metrics under the ischemia-reperfusion paradigm were also found to be correlated with ABI, suggestive of disease severity-dependent impairment of vascular response in PAD patients [5]. Our results suggest that even at rest, vascular function at the tissue level could be indicative of disease progression. However, unlike CMR imaging, ABI cannot be obtained from all patients especially in patients with critical limb ischemia.

In the cuff-induced arterial occlusion experiment on healthy subjects, varying degrees of ischemia were factitiously induced by different pressures above the systolic pressure, and CMR imaging techniques were able to detect these changes. However, in healthy old subjects and PAD patients with differing degrees of ischemia, only BOLD was effective for this discrimination at rest. One possible explanation for this finding could be that the degree of ischemic insult in PAD patients was less severe than that induced by cuff occlusion. Additionally, collateral arteries developed in skeletal muscles in PAD patients would compensate for the perfusion deficit. BOLD is more sensitive to these less dramatic changes, which may be ASL- or IVIM-insensitive.

Numerous previous studies have used ASL or BOLD to monitor dynamic perfusion changes in skeletal muscles at rest and during ischemia and hyperemia, which allows the measurement of key parameters, such as peak hyperemic value (PHV) and time-to-peak (TTP). In our study, we did not measure continuous temporal changes of ASL, BOLD, and IVIM in our subjects mainly because it was not technically feasible to perform these three sequences sequentially at a high temporal resolution. Nevertheless, our protocol can be regarded as a simplified approach to the dynamic scanning method; a similar approach was also used in a previous study [38]. Although PHV and TTP proved useful for the assessment of PAD in most published studies, conflicting results also exist [13]. In addition, the reproducibility of the data is another concern. Versluis et al. investigated the reproducibility of BOLD-derived PHV and TTP values in healthy subjects and PAD patients [39]. The reproducibility was unsatisfying with a coefficient of variation up to 26.7% and an ICC value as low as 0.59 [39]. Moreover, due to massive pain or risk of worsening the clinical condition caused by cuff compression, patients with critical limb ischemia, ulceration, necrosis, or gangrene should be considered with caution or even excluded from the study [40]. Compared with the cuff compression paradigm, the resting-state imaging scheme is simple to perform, less time-consuming, and more acceptable to PAD patients [24]. In our study, we wanted to investigate and were most concerned with whether the baseline measurements of these techniques had value in assessing PAD.

The present study has several limitations. First, the number of study subjects was relatively small. Second, no gold standard for blood flow, oxygenation, or microvascular perfusion in skeletal muscle could be established in our subjects. For example, mixed venous oxygen saturation measurements would be informative regarding the confirmation of BOLD results. Nevertheless, our results revealed significant relationships with TcPO2 measurements, which are commonly used in clinical routines. However, the use of TcPO2 measurement is limited since it is confined to the skin microvasculature and thus fails to directly analyze the skeletal muscle [7]. Therefore, once further validated, noninvasive CMR techniques might be used to gain direct information regarding skeletal muscle perfusion in lower extremities. Third, the cuff compression paradigm was used in the study instead of exercise. Exercise is more physiologically and clinically relevant. A previous study showed that muscle perfusion at peak exercise was correlated with 6-min walk distance in lower extremities [41]. Compared with exercise, cuff compression has improved test-retest reproducibility [42] and less motion artifacts [40]. Moreover, cuff compression allow the assessment of muscles which respond less to commonly used ankle flexion exercise [42]. Lopez et al. suggested the use of cuff compression in a more general study population and the use of exercise in specific PAD therapies in claudicants based on its physiologic and clinical relevance [42]. Fourth, skeletal muscle energetics were not investigated in the current study. ^31^P CMR spectroscopy is a useful tool to noninvasively probe skeletal muscle energetics, including adenosine triphosphate and creatine phosphate metabolism [43], which would help to better understand the perfusion results in our study. Further studies are warranted. Finally, CMR angiography was not performed in the current study.

## Conclusions

In conclusion, the present study shows that multi-parametric CMR techniques including ASL, BOLD, and IVIM provide useful and complementary information regarding tissue perfusion in the lower extremities of healthy subjects. Perfusion-related metrics derived from these techniques correlate with TcPO2 measurements. In PAD patients, BOLD is a more reliable imaging technique for the detection and stratification of alterations in microvascular function at rest compared with ASL and IVIM.

## Additional files

Additional file 1: **Figure S1.** Example of source images and processed maps for arterial spin labeling (ASL), blood-oxygen level dependent (BOLD), and intravoxel incoherent motion (IVIM) cardiovascular magnetic resonance (CMR). (PPTX 11134 kb)

Additional file 2: **Figure S2.** Graphs depicting serial measurements of imaging parameters from ASL, BOLD, and IVIM for the anterior, lateral, soleus, and gastrocnemius muscle groups in the control side in healthy young subjects. (PPTX 727 kb)

Additional file 3: **Figure S3.** Graphs depicting serial measurements of imaging parameters from ASL, BOLD, and IVIM for the anterior, lateral, soleus, and gastrocnemius muscle groups in the control side in healthy old subjects. (PPTX 654 kb)

Additional file 4: **Table S1.** Spearman rank correlation coefficients for ASL, BOLD, and IVIM imaging parameters. (DOCX 16 kb)

Additional file 5: **Table S2.** Comparison of functional imaging parameters between affected and contralateral lower extremities in PAD patients. (DOCX 18 kb)

## Abbreviations

3D: Three dimensional
ABI: Ankle-brachial index
ASL: Arterial spin labeling
BOLD: Blood oxygenation level-dependent
CMR: Cardiovascular magnetic resonance
CT: Computer tomography
ESS: Sum of squared errors
FOV: Field of view
ICC: Intraclass correlation coefficient
IVIM: Intravoxel incoherent motion
PAD: Peripheral arterial disease
PHV: Peak hyperemic value
PLD: Post-labeling delay
ROI: Region of interest
SNR: Signal-to-noise ratio
TcPO2: Transcutaneous oxygen pressure
TE: Echo time
TSS: Sum of squared differences
TTP: Time-to-peak
