# Supplementary material for: Evaluation of skeletal muscle microvascular perfusion of lower extremities by cardiovascular magnetic resonance arterial spin labeling, blood oxygenation level-dependent, and intravoxel incoherent motion techniques
Source: J Cardiovasc Magn Reson. 2018 Mar 19;20:18. doi: 10.1186/s12968-018-0441-3 (PMC5858129; doi:10.1186/s12968-018-0441-3)
Supplement: Supplementary file 1 — Figure S1. Example of source images and processed maps for arterial spin labeling (ASL), blood-oxygen level dependent (BOLD), and intravoxel incoherent motion (IVIM) cardiovascular magnetic resonance (CMR). (PPTX 11134 kb) [file 12968_2018_441_MOESM1_ESM.pptx]

## Slide 1
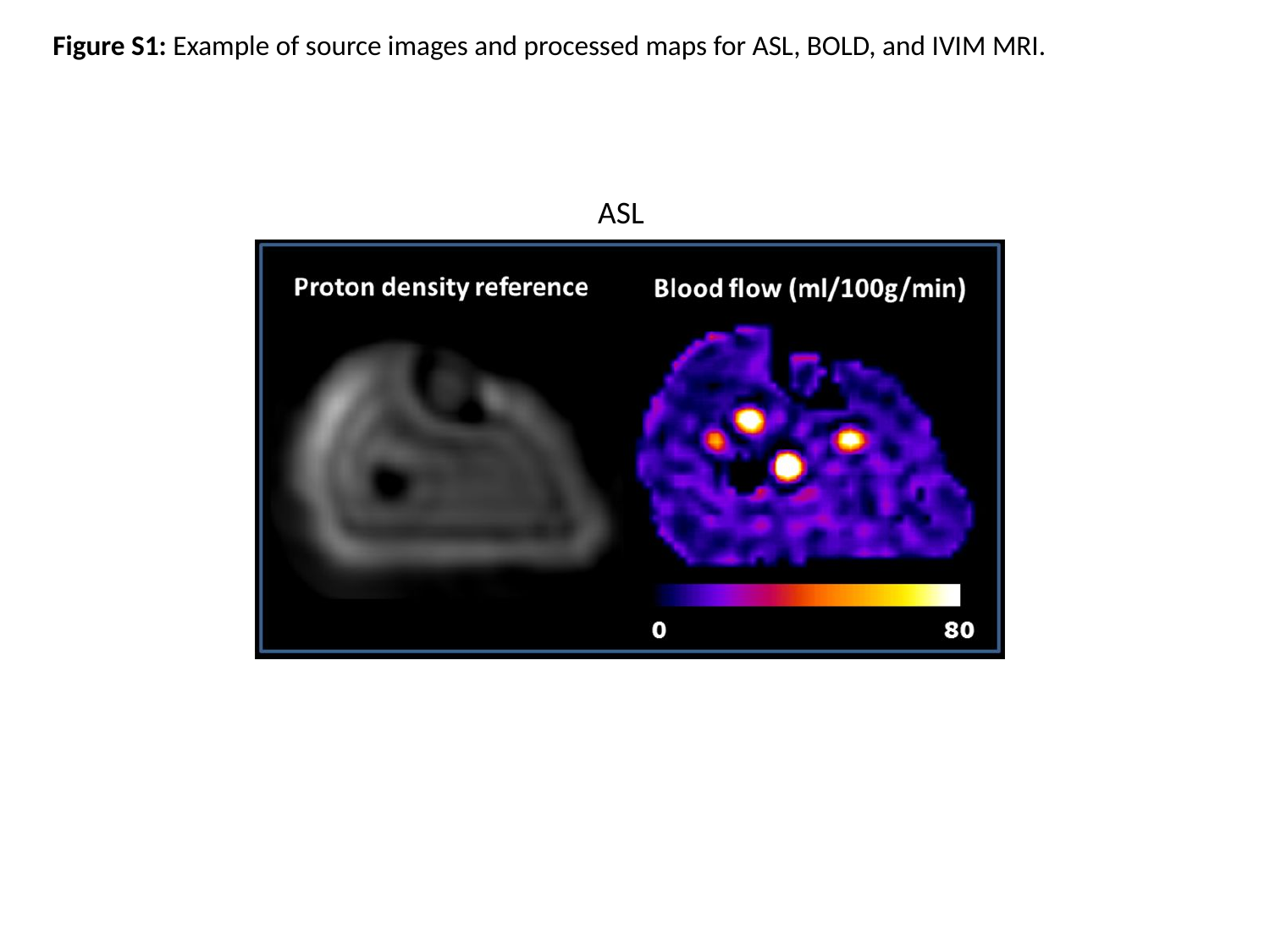

Figure S1: Example of source images and processed maps for ASL, BOLD, and IVIM MRI.
ASL

## Slide 2
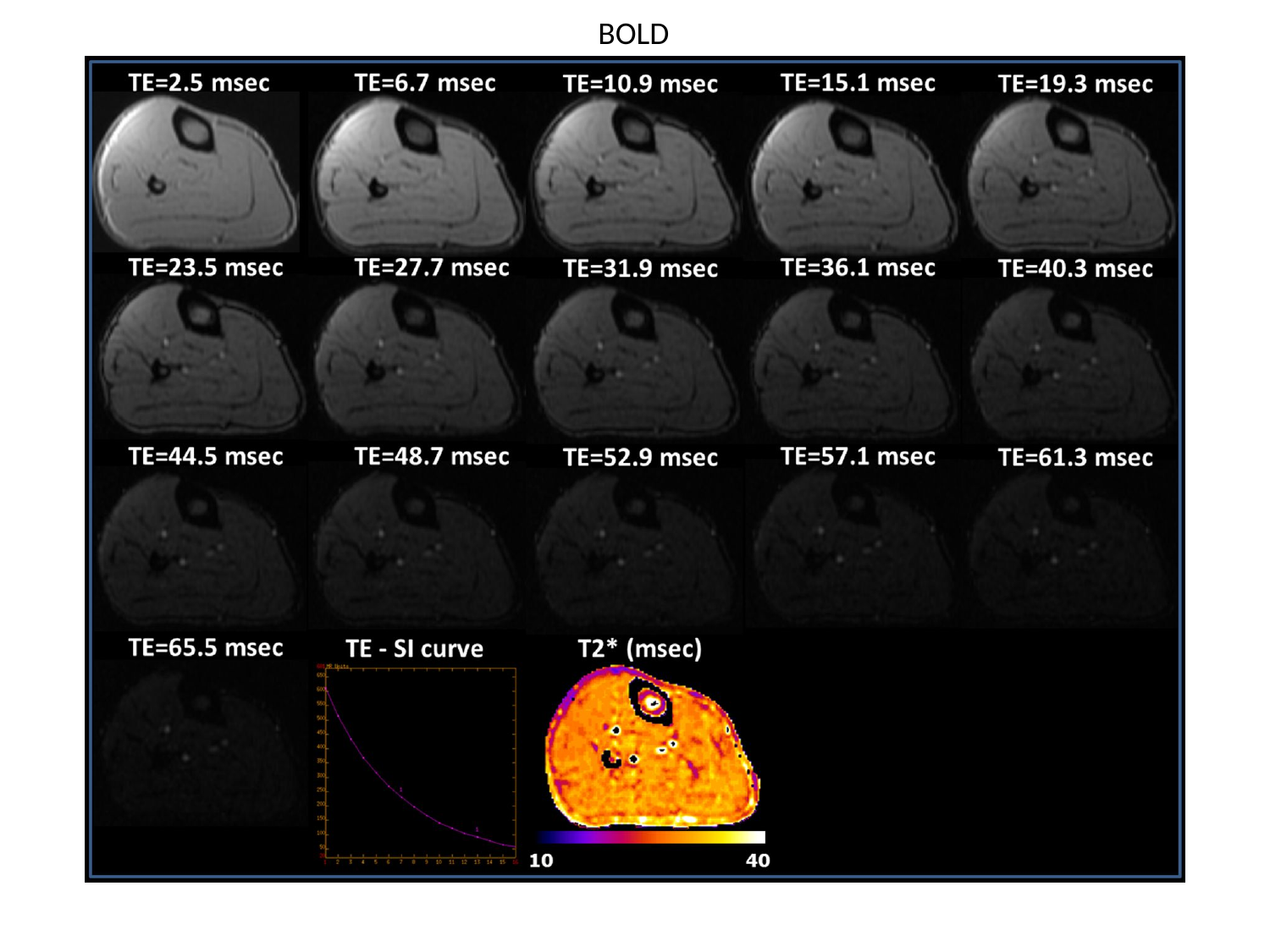

BOLD

## Slide 3
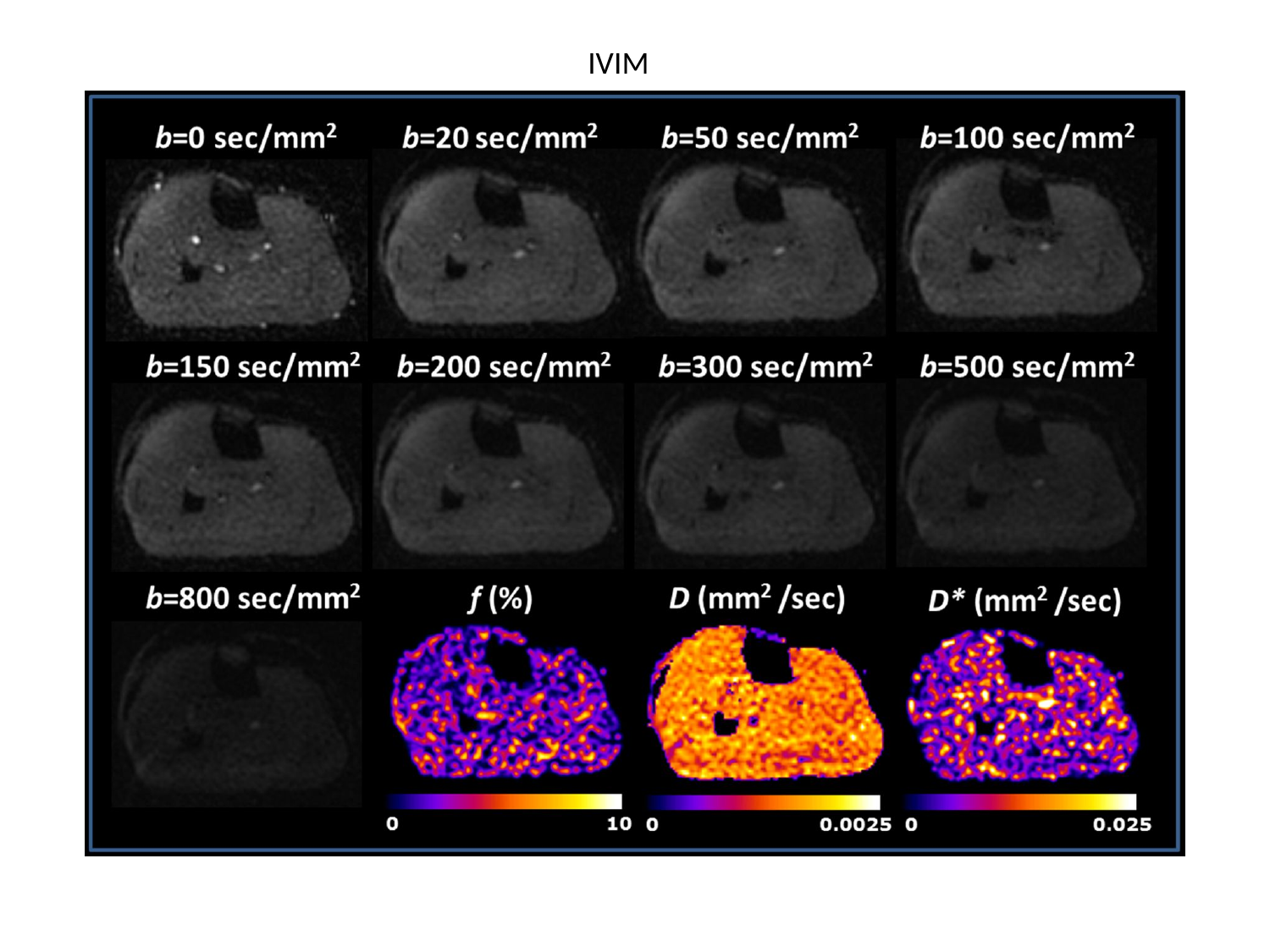

IVIM
