# Supplementary material for: Evaluation of skeletal muscle microvascular perfusion of lower extremities by cardiovascular magnetic resonance arterial spin labeling, blood oxygenation level-dependent, and intravoxel incoherent motion techniques
Source: J Cardiovasc Magn Reson. 2018 Mar 19;20:18. doi: 10.1186/s12968-018-0441-3 (PMC5858129; doi:10.1186/s12968-018-0441-3)
Supplement: Supplementary file 2 — Figure S2. Graphs depicting serial measurements of imaging parameters from ASL, BOLD, and IVIM for the anterior, lateral, soleus, and gastrocnemius muscle groups in the control side in healthy young subjects. (PPTX 727 kb) [file 12968_2018_441_MOESM2_ESM.pptx]

## Slide 1
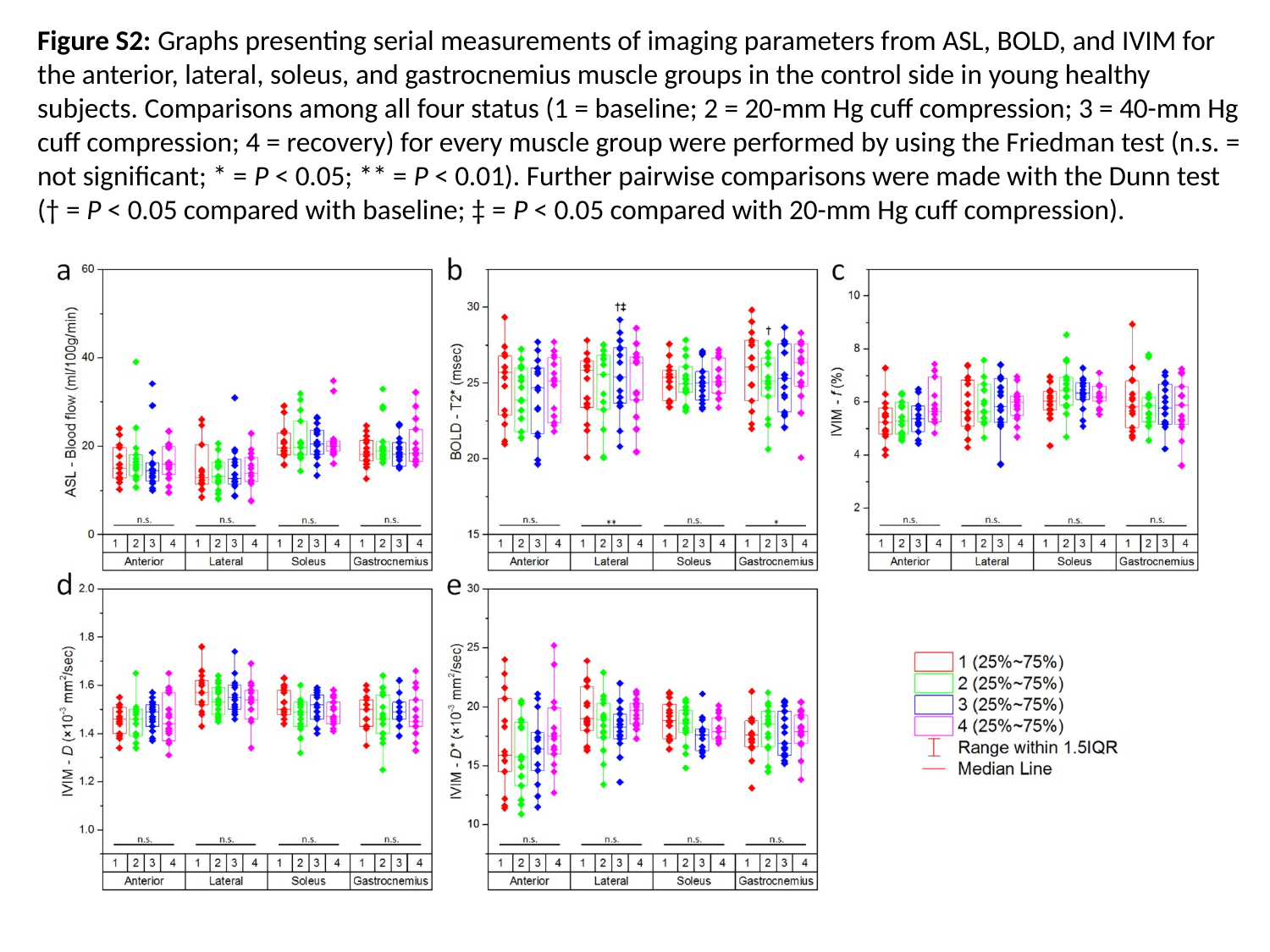

Figure S2: Graphs presenting serial measurements of imaging parameters from ASL, BOLD, and IVIM for the anterior, lateral, soleus, and gastrocnemius muscle groups in the control side in young healthy subjects. Comparisons among all four status (1 = baseline; 2 = 20-mm Hg cuff compression; 3 = 40-mm Hg cuff compression; 4 = recovery) for every muscle group were performed by using the Friedman test (n.s. = not significant; * = P < 0.05; ** = P < 0.01). Further pairwise comparisons were made with the Dunn test († = P < 0.05 compared with baseline; ‡ = P < 0.05 compared with 20-mm Hg cuff compression).
