# Supplementary material for: Evaluation of skeletal muscle microvascular perfusion of lower extremities by cardiovascular magnetic resonance arterial spin labeling, blood oxygenation level-dependent, and intravoxel incoherent motion techniques
Source: J Cardiovasc Magn Reson. 2018 Mar 19;20:18. doi: 10.1186/s12968-018-0441-3 (PMC5858129; doi:10.1186/s12968-018-0441-3)
Supplement: Supplementary file 4 — Table S1. Spearman rank correlation coefficients for ASL, BOLD, and IVIM imaging parameters. (DOCX 16 kb) [file 12968_2018_441_MOESM4_ESM.docx]

**Table S1.**

Spearman rank correlation coefficients for ASL, BOLD, and IVIM imaging parameters.

| Parameter | ASL - Blood flow | BOLD - T2* |
| --- | --- | --- |
| BOLD - T2* | 0.017 (0.895) | … |
| IVIM - *f* | -0.002 (0.986) | -0.055 (0.675) |
| IVIM - *D* | -0.061 (0.645) | 0.034 (0.797) |
| IVIM - *D** | 0.232 (0.074) | -0.017 (0.900) |

Data in parentheses are *P-*values.

ASL = arterial spin labeling; BOLD = blood oxygenation level-dependent; IVIM = intravoxel incoherent motion.
