# Supplementary material for: Evaluation of skeletal muscle microvascular perfusion of lower extremities by cardiovascular magnetic resonance arterial spin labeling, blood oxygenation level-dependent, and intravoxel incoherent motion techniques
Source: J Cardiovasc Magn Reson. 2018 Mar 19;20:18. doi: 10.1186/s12968-018-0441-3 (PMC5858129; doi:10.1186/s12968-018-0441-3)
Supplement: Supplementary file 5 — Table S2. Comparison of functional imaging parameters between affected and contralateral lower extremities in PAD patients. (DOCX 18 kb) [file 12968_2018_441_MOESM5_ESM.docx]

**Table S2.**

Comparison of functional imaging parameters between affected and contralateral lower extremities in PAD patients.

| Parameter | | Affected side | Contralateral side | *P*-value |
| --- | --- | --- | --- | --- |
| ASL-Blood flow (ml/100 g/min) | Anterior | 15.2 (8.1-34.2) | 14.5 (9.3–20.5) | 0.470 |
|  | Lateral | 12.9 (8.2-34.0) | 16.3 (10.1-21.7) | 0.397 |
|  | Soleus | 14.7 (12.0-40.1) | 17.5 (14.5-24.7) | 0.245 |
|  | Gastrocnemius | 14.5 (10.5-25.5) | 15.8 (11.8-22.8) | 0.177 |
| BOLD-T2* (msec) | Anterior | 21.9 (13.4-26.8) | 24.9 (20.2–31.8) | **0.005** |
|  | Lateral | 21.8 (11.5-28.7) | 24.2 (15.9-30.3) | 0.030 |
|  | Soleus | 22.5 (14.8-26.6) | 23.5 (15.7-28.7) | 0.026 |
|  | Gastrocnemius | 22.5 (13.5-26.1) | 24.6 (13.9-29.4) | 0.016 |
| IVIM-*f* (%) | Anterior | 5.7 (4.5-12.0) | 5.1 (4.0–8.8) | 0.124 |
|  | Lateral | 5.6 (4.4-14.5) | 5.3 (3.3-9.6) | 0.286 |
|  | Soleus | 5.2 (3.7-14.2) | 5.5 (2.5-11.0) | 0.331 |
|  | Gastrocnemius | 5.6 (3.7-7.7) | 4.7 (2.0-8.6) | 0.096 |
| IVIM-*D* (×10^-3^ mm^2^ /sec) | Anterior | 1.48 (1.24-2.02) | 1.42 (1.29-1.54) | 0.132 |
|  | Lateral | 1.51 (0.97-1.72) | 1.52 (1.32-1.88) | 0.176 |
|  | Soleus | 1.44 (1.28-1.99) | 1.51 (1.34-2.15) | 0.176 |
|  | Gastrocnemius | 1.51 (1.33-1.88) | 1.57 (1.32-2.01) | 0.463 |
| IVIM-*D** (×10^-3^ mm^2^ /sec) | Anterior | 17.2 (11.0-20.5) | 14.2 (12.4-20.7) | 0.101 |
|  | Lateral | 14.8 (9.1-18.1) | 15.8 (7.7-23.6) | 0.069 |
|  | Soleus | 17.2 (9.4-27.5) | 17.3 (8.4-23.1) | 0.510 |
|  | Gastrocnemius | 17.3 (12.5-21.9) | 18.8 (10.3-22.5) | 0.660 |

Data are medians with ranges in parentheses. After Bonferroni correction, *P* < 0.0125 denotes statistical significance. The statistically significant values are presented in bold.

ASL = arterial spin labeling; BOLD = blood oxygenation level-dependent; IVIM = intravoxel incoherent motion.
